# A Review of Core Concepts of Imaging Informatics

**DOI:** 10.7759/cureus.32828

**Published:** 2022-12-22

**Authors:** Aren Shah, Pooja Sai Muddana, Safwan Halabi

**Affiliations:** 1 College of Literature, Science, and the Arts, University of Michigan, Ann Arbor, USA; 2 Radiodiagnosis, Jawaharlal Institute of Postgraduate Medical Education and Research, Puducherry, IND; 3 Imaging Informatics, Lurie Children’s Hospital, Chicago, USA

**Keywords:** artificial intelligence, workflow, ris, vna, ontologies, hl7, dicom, imaging informatics

## Abstract

There are myriad systems and standards used in imaging informatics. Digital Imaging and Communications in Medicine (DICOM) is the standard for displaying, transferring, and storing medical images. Health Level Seven International (HL7) develops and maintains standards for exchanging, integrating, and sharing medical data. Picture archiving and communication system (PACS) serves as the health provider’s primary tool for viewing and interpreting medical images. Medical imaging depends on the interoperability of several of these systems. From entering the order into the electronic medical record (EMR), several systems receive and share medical data, including the radiology information system (RIS) and hospital information system (HIS). After acquiring an image, transformations may be performed to better focus on a specific area. The workflow from entering the order to receiving the report depends on many systems. Having disaster recovery and business continuity procedures is important should any issues arise. This article intends to review these essential concepts of imaging informatics.

## Introduction and background

System interoperability is required to help facilitate the process from ordering an imaging study to receiving the results [1]. For these systems to communicate, standards are put in place by various organizations. Digital Imaging and Communications in Medicine (DICOM) is a major international standard that describes proper formatting and exchanging of medical images and data [2]. Health Level Seven (HL7) is another international organization that sets standards for exchanging and integrating medical information [3]. In addition to the healthcare technology standards, ontologies provide terms that describe relationships between diagnoses and imaging observations. These standard terms are important as they provide a knowledge model that links diseases and imaging findings.

Before the image is displayed to the radiologist, transformations, including image segmentation, registration, and iterative reconstruction, may be performed [1]. After the image is acquired, the radiologist uses a picture archiving and communication system (PACS) to read and interpret the medical scan. A reading room environment should be equipped with proper workstation configurations to improve efficiency and reduce repetitive strain injuries (RSI) [1]. Recently, artificial intelligence (AI) and machine learning have been leveraged in health care at various points of the imaging information cycle. To help augment the work of a limited number of radiologists, researchers have been exploring the application of AI in radiology to provide faster and more reliable image acquisition and interpretation. 

Radiologists and other healthcare professionals benefit from these existing and emerging technologies as they allow faster and more reliable imaging and interpretation. This review article aims to explore several of the systems and standards used in imaging informatics that radiologists and other practitioners should be familiar with.

## Review

Standards

DICOM

The DICOM is an international standard that outlines the proper storage, display, and transmission of medical images and data [1]. DICOM describes how to properly format and exchange medical images and data for users within and outside the hospital setting [2]. Users across all medical imaging specialties use DICOM as it is the standardization of medical imaging information [2]. The DICOM standard applies to both the pixel-based imaging data and the metadata. The metadata can be found in the “DICOM header,” which has patient information and other facts about the image [1]. Using DICOM reduces possible incorrect data entry as the information about an order is often transmitted between the radiology information system (RIS) and the modality [1]. Since DICOM is an international standard, certain data elements are required to allow network connectivity among multiple systems [2]. Vendors need to provide customers with conformance statements to ensure proper use of the DICOM standard [1].

HL7

The HL7 is an international organization that develops and maintains standards for exchanging, integrating, sharing, and retrieving medical information [1]. HL7 is commonly used to transmit non-image data between multiple systems [1]. Using the electronic health record (EHR) with HL7 allows medical staff to refer to a patient’s medical history when making decisions [3]. Without HL7, there would not be a successful exchange of information and no EHR [3]. 

The HL7 Version 2 (V2) messaging standard facilitates the exchange of medical data across multiple systems [1]. HL7 V2 is considered an international standard and is used in almost every healthcare clinic [4]. One of the reasons for its popularity is that applications can be developed and implemented, allowing an easier exchange of information [4]. Although HL7 Version (V3) is more human-readable, it is less common compared to HL7 V2 because HL7 V3 has increased complexity [1]. Government agencies often use HL7 V3 because it is used in some Integrating the Healthcare Enterprise (IHE) integration profiles [3].

Ontologies

Ontologies refer to the representation of concepts and relationships, which are human-readable and machine computable [5]. Additionally, ontologies are formal terms that define a causal relationship between diagnoses and imaging observations [5]. Automated knowledge discovery and diagnostic reasoning rely on mappings between ontologies [5]. There are several major ontologies, including the Radiological Society of North America (RSNA)’s radiology lexicon (RadLex), Radiology Gamuts Ontology (RGO), and the Systematized Nomenclature of Medicine Clinical Terms (SNOMED CT).

The largest radiology-specific lexicon is RadLex (htttp://radlex.org), which contains thousands of terms that describe imaging anatomy, procedures, and pathology [1]. The RSNA developed RadLex to standardize the clinical vocabulary used in radiological literature, learning materials, and radiology reports [5]. The RadLex Playbook, which is a part of the RadLex ontology, defines imaging exam names and codes [1].

RGO is a formal knowledge model that links diseases and imaging findings with differential diagnosis. RGO consists of 16,918 classes (terms) that represent diseases, interventions, and imaging observations [5]. The ontology has 1782 subclass relations and 55,569 causal relations [5]. RGO consists of highly specific terms, including sloughed calcified renal papilla, which allows one to accurately express differential diagnosis [5].

SNOMED CT is a comprehensive clinical terminology that standardizes clinical content in the electronic medical record (EMR) [5]. Designed to be a US standard for EMR exchange, SNOMED CT contains more than 300,000 terms organized hierarchically [6]. Some hierarchies include body structure, clinical finding, event, procedure, social context, and substance [6]. SNOMED CT consists of four files, including the concept file, description file, relationship file, and hierarchy file [6]. These files identify unique objects, explain the concepts, and show relationships and hierarchical structure [6].

The RGO consists of an interactive reference of radiology gamuts [7]. This ontology includes 1,674 differential diagnoses, 19,017 terms, and 52,976 links between the terms [7]. The differential terms describe imaging findings in both adults and children and observations with multiple modalities [7]. Subclasses have principal classes, which can show the relationship between observations and findings [7].

Reading room environment

PACS

Since the early 2000s, PACS has impacted the design of medical imaging reading environments [8]. The PACS serves as a primary tool for the radiologist to view and interpret medical images [1]. PACS is able to communicate with imaging modalities with DICOM and/or EMR with HL7 transactions [1]. From about 2001 to 2006, most healthcare institutions started to use PACS, which required a physical workstation [9]. With modern PACS being web-based, radiologists have access more easily on mobile devices and desktop computers [1].

Vendor-Neutral Archive

PACS uses an archive to ensure that medical records and documents are stored for future access. When new PACS vendors are used, data previously stored need to be migrated to the new systems [8]. Through the migration of data, medical records and documents can easily be transferred from old PACS to new systems [8]. The vendor-neutral archive (VNA) helps ease the transition by storing data in a central archive [1]. The application engine receives and integrates data using a different DICOM syntax [8]. The VNA allows users to access several DICOM images and non-DICOM data [1]. In enterprise imaging, handling metadata with a non-DICOM image is still a major challenge [1].

RIS

The RIS helps manage several aspects of an imaging exam. The RIS contains scheduling information, tracking, result notification, and billing information [1]. Some institutions use the RIS as a standalone application and others use RIS as part of the EMR [1].

Image Displays

Images need to be presented consistently to technologists, radiologists, and other physicians. Spatial and contrast resolutions of images need to be properly displayed for accurate interpretations. The way the image is presented is impacted by the workstation software, graphic controllers, and display devices [10]. Below, we review some common characteristics of image displays.

Workstation display monitors are separated into diagnostic displays, primary interpretation displays, and non-diagnostic displays [10].

Graphic bit depth: Most operating systems of workstations have 8 bits (256 values) that manage images with red, green, and blue channels. There are 256 available gray levels where the values of red, green, and blue are equal. Some operating systems of workstations have 30-bit graphics with 10 bits per channel, which requires them to need support from the operating system, workstation software, graphics card, and monitor [10]. Some differences exist between the 8-bit and 10-bit systems, but diagnostic interpretations have not been shown to be affected [10].

Display technology: Most workstations use liquid crystal display (LCD) or organic light-emitting diode (OLED) panels, which provide very good resolution without significant distortion [10]. Flat panel surfaces minimize reflections and glare by absorbing ambient light. Some lower-cost LCD units use twisted nematic (TN) pixel structures. These units should not be used for medical image viewing because the brightness, contrast, and color are all severely altered [10]. Other LCD pixel structures offer better viewing angle performance, which needs to be evaluated when purchasing displays for medical image viewing [10].

Graphic interface: LCD and OLED have an internal buffer storing data for each pixel of the device. The transfer of image data to a digital format uses the interface between the graphic controller and the LCD device. Digital formats that hold the image data include DVI-D and DisplayPort [10]. Setting the graphic controller device driver to native rows and columns can help display optimal resolution [10]. Conversions between digital to analog and vice versa are strongly discouraged in the LCD device as they can result in image degradation [10]. 

Image presentation size: The application software and the graphic controller work together to use the acquired image data to get the displayed image data [10]. The size of the rows and columns of the displayed image is slightly different from that of the actual acquired image [10]. To improve image resolution, the interpolation of each pixel should consider more than the closest four acquired pixel values [10]. Each displayed pixel can be interpolated using up-sampling or down-sampling. Other interpolation algorithms used with the graphic controller include cubic spline and cubic polynomial interpolation. These methods present quality images with negligible decay [10].

Compression

Compression is used to decrease the file size of an image, which speeds up file transfer time and reduces the storage requirements of the file [1]. Users can compress files without losing important information by decreasing redundant image information, such as the backgrounds of images [1]. Reversible compression reduces file sizes by about 3:1, which saves space while also preserving the image content [1]. Since reversible compression has no impact on the image, it may always be used [10]. When discarding unnecessary or minimally important image information, irreversible compression reduces file sizes by about 10:1 [1]. Irreversible compression cannot always be used because there needs to be sufficient image content to perform a clinical task [10]. Although irreversible compression reduces file transmission time, it can only be done in certain circumstances [10].

Speech Recognition

Speech recognition (SR) converts speech to words in written text and has been used in medicine for several decades. Advances in SR have resulted in it being used to produce radiology reports. Practices that do not use SR have transcriptionists that type the dictated voice. The process of creating a report starts when a radiologist dictates a case [11]. The transcriptionist types out the dictation and sends the report back to the radiologist to review it [11]. The radiologist can review the text and correct any errors, which are often due to background noise, mistranscribed words, omitted words, and words not recognized by SR technology [12]. After the radiologist edits or accepts the report, clinicians can review the report [11]. Without SR technology, there can often be a delay between the transcriptionist and the radiologist [11].

By using SR technology, the radiologist can dictate the case and immediately edit or accept it [11]. Using SR reduces the amount of time it takes to allow the report to flow to EMR, making the results available to clinicians [11]. SR reduces the report production time, reduces staffing needs, and decreases cost [11]. RSNA and other organizations are working to improve SR technology by improving consistency in reporting templates and common data elements [12]. Due to these benefits, radiology departments increasingly use SR technology rather than traditional transcription services [12]. 

Ergonomics

Since radiologists spend several hours working on a computer, they are susceptible to RSI, including carpal and cubital tunnel syndrome [1]. Carpal tunnel syndrome can occur due to dorsiflexion of the wrist while typing [1]. Cubital tunnel syndrome impacts the wrist or the elbow [1]. Due to possible RSIs, it is important to have workstation configurations that promote healthy body positions and appropriate distance between the user and the display [1]. 

Reading areas should have adequate airflow, optimal temperatures, and humidity control [10]. Studies show that radiologists are more productive in cooler rooms [9]. The optimal temperature for the reading room is about 72 degrees Fahrenheit [9]. Reading areas should set ambient lighting to minimize specular and diffuse reflection on the display [10]. Additionally, ambient lighting can minimize eye strain [1].

Other considerations include noise level, proper furniture, and reference tools. In the reading room, noise needs to be minimized in order to allow the radiologist to concentrate [10]. In reading rooms, proper furniture is essential to allow productivity. Chairs with lumbar support and height controls can reduce fatigue and injuries. Adjustable workstation tables are often more comfortable and allow greater efficiency. Additionally, the table needs to be positioned so that the viewer is an arm’s length away from the display (⅔ m or 60 cm) [10]. During image interpretation, reference and dictation tools need to be easily accessible [10].

Workflow considerations

Workflow Steps

**Figure 1:**
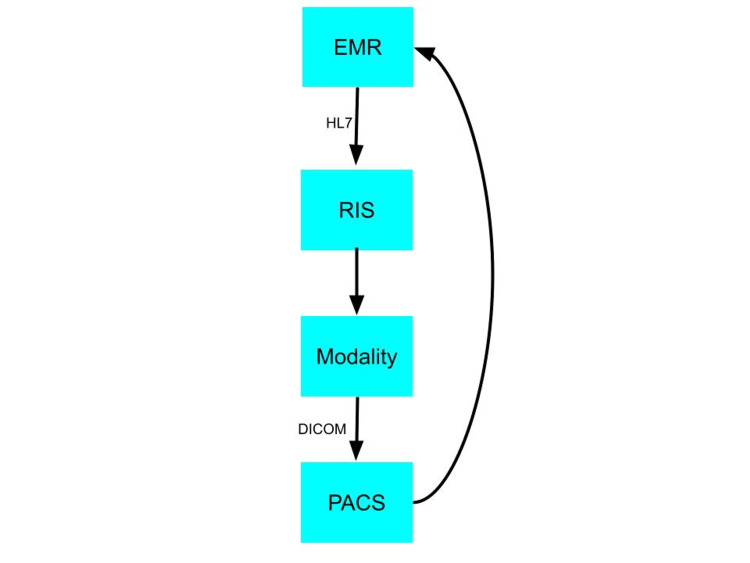
Flow of Imaging Information EMR, electronic medical record; HL7, Health Level Seven; RIS, radiology information system; DICOM, Digital Imaging and Communications in Medicine; PACS, picture archiving and communication system.

From the time a physician orders an imaging scan to the report, several systems, including PACS, RIS, and EMR, work interoperably [1]. When a physician decides to order an imaging scan for a patient, the order is placed in EMR [1]. After the order is placed, HL7 transactions communicate the order to the RIS [1]. The RIS then sends the order information to the appropriate modality via the DICOM Modality Work List [1]. Afterward, the modality communicates with the PACS via DICOM transactions [1]. Figure 1 is a flowchart of the imaging information. The radiologist can view and dictate the report using voice recognition [1]. After the radiologist is finished with the image report, the reporting software sends the report to the RIS and EMR via HL7 transactions [1]. Using EMR requires the proper exchange of information between several systems [3]. This exchange cannot occur without HL7 transactions [3]. This technology allows proper observation reporting, order management, and patient management information [3]. 

Downtime Procedures

Disaster recovery (DR) and business continuity (BC) are two common downtime procedures [1]. DR procedures occur after a large-scale, unexpected, and disruptive event [1]. These events could be due to a failure in hardware or software, which can interrupt the workflow. Some of the most frequent causes of downtime include planned downtime, application failure, operator error, and operation system failure [13]. These interruptions can impact patient care. Therefore, large-scale PACS must have a DR plan to secure data [13]. Radiologists cannot operate when there is a disruption and loss of the IT infrastructure [13]. Therefore, having a DR plan is very important [13]. DR policies usually include off-site data backup systems and steps to recover critical data [1]. BC policies usually include systematic precautions and backups needed to continue to care for a patient while there is a system failure [1].

The medical imaging systems that radiologists use are considered high-availability (HA) systems, which means that they can perform automated recovery and failover operations during a disruption [1]. When there is a description in the system, clustering ensures that the process is switched to another machine [13]. This failover in the cluster does not cause a disruption, which makes it a HA system that maintains data accessible during unexpected events [13]. Additionally, medical imaging systems have a fault tolerance (FT), which refers to the ability of a system to function when a component fails [1]. Medical imaging systems have a very high FT because there are several identical components, making it less likely for the system to stop functioning [1].

Teleradiology

Teleradiology refers to the practice of radiologists interpreting medical images remotely. Teleradiologists should have relevant clinical information, including previous imaging examinations, EMRs, and relevant medical history [14]. It can often be difficult for teleradiologists to access such information as they often interpret medical images for multiple institutions, which may have different PACS and EMR systems [14]. Further integration of PACS and EMR systems can reduce these barriers that teleradiologists may face [14]. The rise of teleradiology in the past decades has expanded access to imaging in rural and underserved communities. Many rural regions have few radiologists and even less specialized radiologists. Disparities in these communities have been partly addressed through teleradiology.

Data privacy and security

De-identification of Images

To protect others’ privacy, it is important to remove protected health information (PHI) from the imaging examination so that the identity of the patient cannot be determined [1]. De-identified images can contain information that can allow an approved organization to identify a patient [1]. When personal information from an imaging examination is anonymized, all PHI and other identifiable data are removed. Therefore, the patient’s identity is unable to be revealed and cannot be found later [1]. PHI in metadata can usually be removed, but sometimes “burned-in” PHI must also be removed to de-identify images [1]. Additionally, PHI includes the face of the patient as his or her face can be reconstructed using an image [1].

De-identification of Reported Text

De-identifying report data require more careful review compared to de-identifying image data [1]. PHI is not usually found in a radiology report text, so de-identifying report text usually requires manual review or special algorithms [1]. These specialized algorithms do not always work too well because of the difference in frequencies of PHI in radiology report text compared to medical text [1].

Image Post-processing

After an image is captured, transformations are performed to an image through the process of “post-processing” [1]. Many practices perform image post-processing in three-dimensional (3D) visualization labs. Many of these labs are in academic radiology departments [15]. 

Post-processing often occurs before the image is displayed and interpreted. There are several post-processing techniques, including segmentation, registration, and reconstruction [1]. One of the post-processing techniques is image segmentation, which consists of isolating a specific anatomical structure in a medical image [16]. For example, segmentation algorithms may be used to isolate malignant lesions in mammograms [16]. Each segmentation can vary based on the anatomy and imaging modality [16].

Another post-processing technique is image registration, which involves processing information from multiple images. This technique consists of aligning images in corresponding locations to allow a direct comparison [16]. There are several practical applications for image registration. There may be images with different modalities from the same subject that need to be compared [16]. The patient’s physician may want to compare images from the same patient taken at different points in time to track progress [16]. In these situations, image registration allows multiple images to be compared [16].

Iterative reconstruction reconstructs raw CT sinogram data into actual image data. By performing several rounds of image reconstruction, the signal is improved and the noise is reduced [1]. Fewer noise results in less radiation being used to acquire the images, which ultimately decreases the patient’s radiation exposure [1].

Image Artificial Intelligence

AI has demonstrated progress in image-recognition tasks. AI is a growing topic of computer science with the goal of mimicking human intelligence [17]. Within health care, AI is being used in drug recovery, patient monitoring, medical diagnostics, risk management, and more [17].

The goal of AI in medical imaging is to increase efficacy and efficiency, especially because of the disproportionate rate of trained radiologists [17]. Healthcare providers are looking for ways to increase productivity due to a decline in imaging reimbursements [17]. Radiologists have large workloads and using AI may improve efficiency and efficacy. Due to large workloads, average radiologists interpret one image every 3-4 seconds in an 8-hour workday [17]. Due to constrained conditions, AI may be able to reduce errors and improve efficiency [17]. Significant efforts are being made to advance the use of AI in medical imaging.

## Conclusions

The interoperability between the systems is important to a smooth workflow. The standards across each system help facilitate their interactions, increasing efficiency. DR and BC procedures are important should unexpected events arise. AI has already been used in clinical settings. In radiology, AI is still being researched as it has the potential to reduce errors and improve efficiency. To understand the imaging workflow, radiologists should be familiar with the core concepts of imaging informatics.
